# Mathematical Modelling of Human African Trypanosomiasis Using Control Measures

**DOI:** 10.1155/2018/5293568

**Published:** 2018-11-22

**Authors:** Hamenyimana Emanuel Gervas, Nicholas Kwasi-Do Ohene Opoku, Shamsuddeen Ibrahim

**Affiliations:** ^1^African Institute for Mathematical Sciences, Biriwa, Cape Coast, Ghana; ^2^University of Dar es Salaam, Dar es Salaam, Tanzania; ^3^University of Dodoma, Dodoma, Tanzania; ^4^University of Cape Coast, Cape Coast, Ghana

## Abstract

Human African trypanosomiasis (HAT), commonly known as sleeping sickness, is a neglected tropical vector-borne disease caused by trypanosome protozoa. It is transmitted by bites of infected tsetse fly. In this paper, we first present the vector-host model which describes the general transmission dynamics of HAT. In the tsetse fly population, the HAT is modelled by three compartments, while in the human population, the HAT is modelled by four compartments. The next-generation matrix approach is used to derive the basic reproduction number, *R*_0_, and it is also proved that if *R*_0_ ≤ 1, the disease-free equilibrium is globally asymptotically stable, which means the disease dies out. The disease persists in the population if the value of *R*_0_ > 1. Furthermore, the optimal control model is determined by using the Pontryagin's maximum principle, with control measures such as education, treatment, and insecticides used to optimize the objective function. The model simulations confirm that the use of the three control measures is very efficient and effective to eliminate HAT in Africa.

## 1. Introduction

Human African trypanosomiasis (HAT), commonly known as sleeping sickness, is a vector-borne tropical disease which is caused by *Trypanosoma brucei* protozoa species. It is one of the neglected tropical diseases which affect people in sub-Saharan Africa, specifically those living in rural areas. HAT is caused by two species of protozoa which are *Trypanosoma brucei gambiense* (TBG), which causes the chronic form of HAT in central and western Africa, and *Trypanosoma brucei rhodesiense* (TBR), which causes the acute form of the disease in eastern and southern Africa [1]. The HAT disease has killed millions of people since the beginning of 20^*th*^ century and it is transmitted from one individual to another by tsetse flies (genus *Glossina*); TBG is transmitted by riverine tsetse species, while TBR is transmitted by savanna tsetse species [1]. Rhodesiense HAT is an acute disease that can lead to death if not treated within 6 months, while gambiense HAT is a slow chronic progressive disease which causes death with an average duration of 3 years [2]. The signs and symptoms for both forms of HAT are not specific and their appearances vary from one person to another; at the first stage of HAT, the disease is not severe and the signs and symptoms such as intermittent fever, headache, pruritus, lymphadenopathies, asthenia, anemia, cardiac disorders, endocrine disturbances, musculoskeletal pains, and hepatosplenomegaly may be observed, while in the second stage of HAT, sleep disorders and neuropsychiatric disorders are likely to dominate. The HAT disease can be treated by using drugs such as suramin, eflornithine, melarsoprol, and pentamidine.

The disease is reported to affect about 37 sub-Saharan African countries; it affects much rural areas where there are suitable environments for the tsetse flies to live and reproduce, and the periurban areas can also be affected. The transmission of HAT can occur during human activities such as hunting, farming, as well as fishing [3]. The transmission of HAT needs the reservoir; *reservoir* is a species that can permanently maintain the pathogen and from which the pathogen can be transmitted to the target population [4]. Rhodesiense HAT is zoonotic which requires a nonhuman reservoir (animals) for maintaining its population, while in gambiense HAT, humans act as key reservoir [4].

Mathematical models have been used to study the transmission and effective control of diseases simply and cheaply with no need of expensive and complicated experiments [5]. So far, different models have been developed and formulated by different researchers. One of the important modelling work on HAT was done by Rogers [6]; the model explained the mathematical framework on transmission of HAT in multiple host populations [6]. Rogers' model was generalized by Hargrove et al [7], and a new parameter which allows the tsetse flies to feed off multiple hosts was introduced. The model compared the effectiveness of two methods used to control HAT: insecticide-treated cattle and the use of trypanocide drugs to treat cattle. They found out that treating cattle with insecticides is more effective and a cheaper approach to control HAT than using trypanocide drugs. Kajunguri [8] developed a model which was based on a constant population with a fixed number of domestic animals, human, and tsetse flies in one of the villages in West Africa. The major findings of their model estimated that the cattle population contributes to about 92% of the total TBR transmission, while the rest 8% is the contribution of human population in transmission of the disease. The study by Kajunguri [8], which also formulated a multihost model, was used to study the control of tsetse flies and TBR in southern Uganda. They found out that the effective application of insecticides brings about a cost-effective method of control and eliminating the disease. They realized that using insecticides for controlling HAT is more effective and efficient in the area where there are few wild hosts.

Due to low mortality rate of the disease and poverty of its sufferers, the efforts toward the control of HAT has reduced. Most attention is given to popular diseases such as HIV/AIDS, tuberculosis, malaria, and ebola, although the disease is still a threat to the lives of sub-Saharan African people. Moreover, very few studies have been carried out on applying optimal control theory to HAT transmission models. In this paper, we use optimal control theory to study the transmission dynamics of HAT diseases by using education, treatment, and insecticides as the control measures.

The rest of this paper is outlined as follows: Section 2 represents the vector-host model and the underlying assumptions. In section 3, the model equilibria and stabilities are determined, whereas in Section 4, the optimal control model is analyzed by modifying the previous one to control the HAT by using control measures (education, insecticides, and treatment). In addition, the numerical simulations for the optimal control model are done in this section and we use the results obtained to compare the efforts of each control measure to control the HAT in Africa. Finally, we provide the conclusion in Section 5.

## 2. Model Formulation

In this section, the vector-host model as well as the necessary differential equations to describe the transmission of HAT from tsetse fly to human and vice versa are developed. The transmission of HAT in the human population is modelled using four subclasses: Susceptible *S*_H_, Exposed *E*_H_, Infectious *I*_H_, and Recovered *R*_H_. The total human population, *N*_H_, is thus defined by

(1) $$\begin{array}{c} N_{\text{H}}=S_{\text{H}}+E_{\text{H}}+I_{\text{H}}+R_{\text{H}}. \end{array}$$

The transmission of HAT in the vector (tsetse flies) population is also divided into Susceptible (*S*_V_), Exposed (*E*_V_), and Infectious (*I*_V_). The total population of the tsetse flies, *N*_V_, is also defined by

(2) $$\begin{array}{c} N_{\text{V}}=S_{\text{V}}+E_{\text{V}}+I_{\text{V}}. \end{array}$$

We assume a constant population for both host and vector. It is also assumed that the tsetse fly cannot recover from the disease and the infected tsetse fly remains infectious throughout the rest of its life; there is no disease-induced death rate for tsetse flies and the recruitment rates are assumed to be constant due to birth and immigration.

In our model, the recruitment rate of hosts and vectors are represented by *π*_H_ and *π*_V_, respectively. The susceptible host gets the disease when bitten by infectious tsetse fly, and susceptible tsetse fly gets the disease when it bites an infectious human at the rate *a*. The natural mortality rate for humans and vectors are represented by *μ*_H_ and *μ*_V_, respectively. The parameter *ω* represents the disease-induced death rate for humans, while *ξ*_H_ and *ξ*_V_ are the force of infection for humans and vectors, respectively. The parameter *σ* represents per capita rate of a vector becoming infectious, and the rest of the parameters are explained in Table 1. Assuming that the transmission per bite from infectious tsetse fly to human is *a*, then the rate of infection per susceptible human is given by

(3) $$\begin{array}{c} \xi_{\text{H}}=\frac{ap_{\text{H}}I_{\text{V}}}{N_{\text{V}}}, \end{array}$$

and also if we further assume that *a* is the tsetse-fly biting rate, that is, the average number of bites per tsetse fly per unit, then the rate of infection per susceptible tsetse fly can be represented by

(4) $$\begin{array}{c} \xi_{\text{V}}=\frac{ap_{\text{V}}I_{\text{H}}}{N_{\text{H}}}. \end{array}$$

**Figure 1:**
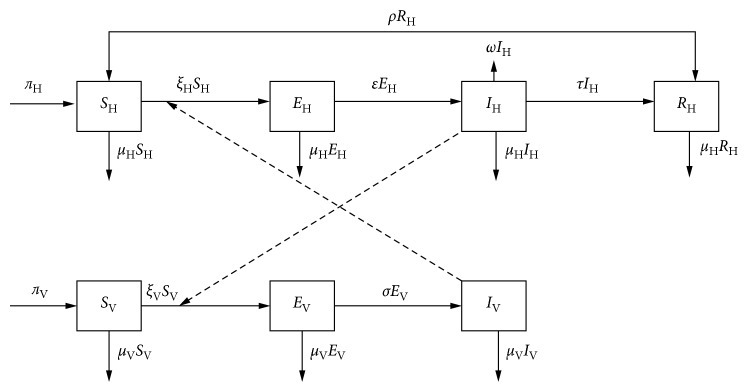
Compartmental model for the transmission of human African trypanosomiasis.

From the model diagram in Figure 1, the following differential equations are derived:

(5) $$\begin{array}{c} \left\{\begin{array}{c} \frac{dS_{\text{H}}}{dt}=\pi_{\text{H}}N_{\text{H}}+\rho R_{\text{H}}-\frac{ap_{\text{H}}I_{\text{V}}}{N_{\text{V}}}S_{\text{H}}-\mu_{\text{H}}S_{\text{H}},\\ \frac{dE_{\text{H}}}{dt}=\frac{ap_{\text{H}}I_{\text{V}}}{N_{\text{V}}}S_{\text{H}}-\varepsilon E_{\text{H}}-\mu_{\text{H}}E_{\text{H}},\\ \frac{dI_{\text{H}}}{dt}=\varepsilon E_{\text{H}}-\mu_{\text{H}}I_{\text{H}}-\omega I_{\text{H}}-\tau I_{\text{H}},\\ \frac{dR_{\text{H}}}{dt}=\tau I_{\text{H}}-\rho R_{\text{H}}-\mu_{E}R_{\text{H}},\\ \frac{dS_{\text{V}}}{dt}=\pi_{\text{V}}N_{\text{V}}-\mu_{\text{V}}S_{\text{V}}-\frac{ap_{\text{V}}I_{\text{H}}}{N_{\text{H}}}S_{\text{V}},\\ \frac{dE_{\text{V}}}{dt}=\frac{ap_{\text{V}}I_{\text{H}}}{N_{\text{H}}}S_{\text{V}}-\mu_{\text{V}}E_{\text{V}}-\sigma E_{\text{V}},\\ \frac{dI_{\text{V}}}{dt}=\sigma E_{\text{V}}-\mu_{\text{V}}I_{\text{V}}. \end{array}\right. \end{array}$$

From system (5), the dimensionless technique is used to derive another equivalent differential equation; we denote *s*_h_=(*S*_H_/*N*_H_), *e*_h_=(*E*_H_/*N*_H_), *i*_h_=(*I*_H_/*N*_H_), *r*_h_=(*R*_H_/*N*_H_), *s*_v_=(*S*_V_/*N*_V_), *e*_v_=(*E*_V_/*N*_V_), and *i*_v_=(*I*_V_/*N*_V_) and substitute in system (5), to obtain the following new equivalent equations:

(6) $$\begin{array}{c} \left\{\begin{array}{c} \frac{ds_{\text{h}}}{dt}=\pi_{\text{h}}+\rho r_{\text{h}}-ap_{\text{h}}i_{\text{v}}s_{\text{h}}-\mu_{\text{h}}s_{\text{h}},\\ \frac{de_{\text{h}}}{dt}=ap_{\text{h}}i_{\text{v}}s_{\text{h}}-\varepsilon e_{\text{h}}-\mu_{\text{h}}e_{\text{h}},\\ \frac{di_{\text{h}}}{dt}=\varepsilon e_{\text{h}}-\mu_{\text{h}}i_{\text{h}}-\omega i_{\text{h}}-\tau i_{\text{h}},\\ \frac{dr_{\text{h}}}{dt}=\tau i_{\text{h}}-\rho r_{\text{h}}-\mu_{\text{h}}r_{\text{h}},\\ \frac{ds_{\text{v}}}{dt}=\pi_{\text{v}}-\mu_{\text{v}}s_{\text{v}}-ap_{\text{v}}i_{\text{h}}s_{\text{v}},\\ \frac{de_{\text{v}}}{dt}=ap_{\text{v}}i_{\text{h}}s_{\text{v}}-\mu_{\text{v}}e_{\text{v}}-\sigma e_{\text{v}},\\ \frac{di_{\text{v}}}{dt}=\sigma e_{\text{v}}-\mu_{\text{v}}i_{\text{v}}. \end{array}\right. \end{array}$$

Table 1 shows the description of the model parameters and variables.

### 2.1. Positivity and Boundedness of the Solutions

In this subsection, we show that system (6) is epidemiologically and mathematically well defined in the positive invariant region:

(7) $$\begin{array}{c} D=\left\{\left(s_{\text{h}},e_{\text{h}},i_{\text{h}},r_{\text{h}},s_{\text{v}},e_{\text{v}},i_{\text{v}}\right)\in R_{+}^{7}:n_{\text{h}}\leq \frac{\pi_{\text{h}}}{\mu_{\text{h}}};n_{\text{v}}\leq \frac{\pi_{\text{v}}}{\mu_{\text{v}}}\right\}. \end{array}$$

**Theorem 1 .**

There exists a domain *D* in which the solution (*s*_h_, *e*_h_, *i*_h_, *r*_h_, *s*_v_, *e*_v_, *i*_v_) is contained and bounded.

*Proof*. We provide the proof following the idea by Olaniyi and Obabiyi [9]. Given the solution set (*s*_h_, *e*_h_, *i*_h_, *r*_h_, *s*_v_, *e*_v_, *i*_v_) with the positive initial conditions (*s*_h_0__, *e*_h_0__, *i*_h_0__, *r*_h_0__, *s*_v_0__, *e*_v_0__, *i*_v_0__), we define

(8) $$\begin{array}{c} n_{\text{h}}\left(s_{\text{h}},e_{\text{h}},i_{\text{h}},r_{\text{h}}\right)=s_{\text{h}}\left(t\right)+e_{\text{h}}\left(t\right)+i_{\text{h}}\left(t\right)+r_{\text{h}}\left(t\right)\text{ and}\\ n_{\text{v}}\left(s_{\text{v}},e_{\text{v}},i_{\text{v}}\right)=s_{\text{v}}\left(t\right)+e_{\text{v}}\left(t\right)+i_{\text{v}}\left(t\right). \end{array}$$

The derivatives of *n*_h_ and *n*_v_ with respect to time along the solution of system (6) for human and tsetse flies, respectively, are obtained by

(9) $$\begin{array}{c} n_{\text{h}}^{\prime }=\frac{ds_{\text{h}}}{dt}+\frac{de_{\text{h}}}{dt}+\frac{di_{\text{h}}}{dt}+\frac{dr_{\text{h}}}{dt}\\ =\pi_{\text{h}}-\left(s_{\text{h}}+e_{\text{h}}+i_{\text{h}}+r_{\text{h}}\right)\mu_{\text{h}}-\omega i_{\text{h}}\\ =\pi_{\text{h}}-n_{\text{h}}\mu_{\text{h}}-\omega i_{\text{h}},\\ n_{\text{v}}^{\prime }=\frac{ds_{\text{v}}}{dt}+\frac{de_{\text{v}}}{dt}+\frac{di_{\text{v}}}{dt}\\ =\pi_{\text{v}}-\left(s_{\text{v}}+e_{\text{v}}+i_{\text{v}}\right)\mu_{\text{v}}\\ =\pi_{\text{v}}-n_{\text{v}}\mu_{\text{v}}. \end{array}$$

From these differential equations, it follows that *n*_h_′ ≤ *π*_h_ − *μ*_h_ *n*_h_ and *n*_v_′ ≤ *π*_v_ − *μ*_v_ *n*_v_. We obtain the solutions as follows:

(10) $$\begin{array}{c} n_{\text{h}}\leq \frac{\pi_{\text{h}}}{\mu_{\text{h}}}\left(1-\exp\left(-\mu_{\text{h}}t\right)\right)+n_{\text{h}}\left(s_{{\text{h}}_{0}},e_{{\text{h}}_{0}},i_{{\text{h}}_{0}},r_{{\text{h}}_{0}}\right)\exp\left(-\mu_{\text{h}}t\right),\\ n_{\text{v}}\leq \frac{\pi_{\text{v}}}{\mu_{\text{v}}}\left(1-\exp\left(-\mu_{\text{v}}t\right)\right)+n_{\text{v}}\left(s_{{\text{v}}_{0}},e_{{\text{v}}_{0}},i_{{\text{v}}_{0}}\right)\exp\left(-\mu_{\text{v}}t\right). \end{array}$$

By taking the limits of both *n*_h_ and *n*_v_ above as *t*⟶*∞*, we obtain *n*_h_ ≤ (*π*_h_/*μ*_h_) and *n*_v_ ≤ (*π*_v_/*μ*_v_); hence, the solutions are contained in the region *D*. This implies that all solutions of the human and tsetse fly population are contained in the region *D* and are nonnegative; this guarantees that the positive invariant region for system (6) exists and is given by

(11) $$\begin{array}{c} D=\left\{\left(s_{\text{h}},e_{\text{h}},i_{\text{h}},r_{\text{h}},s_{\text{v}},e_{\text{v}},i_{\text{v}}\right)\in R_{+}^{7}:n_{\text{h}}\leq \frac{\pi_{\text{h}}}{\mu_{\text{h}}};n_{\text{v}}\leq \frac{\pi_{\text{v}}}{\mu_{\text{v}}}\right\}. \end{array}$$

## 3. Model Equilibria and Stability Analysis

In this section, we give the model equilibria, the basic reproduction number, *R*_0_, and the stabilities at both disease-free and endemic equilibrium.

### 3.1. Disease-Free Equilibrium (DFE)

The DFE in system (6) is when there are no HAT infections within the human and tsetse fly population. Thus, the existence of the DFE is given by *E*_0_=((*π*_h_/*μ*_h_), 0, 0, 0, (*π*_v_/*μ*_v_), 0, 0).

### 3.2. Endemic Equilibrium (EE)

The EE is the nontrivial equilibrium point at which the HAT disease persists in both human and tsetse fly population. Thus, the EE is obtained as follows: *E*_*∗*_=(*s*_h_^*∗*^, *e*_h_^*∗*^, *i*_h_^*∗*^, *r*_h_^*∗*^, *s*_v_^*∗*^, *e*_v_^*∗*^, *i*_v_^*∗*^), where

(12) $$\begin{array}{c} \left\{\begin{array}{c} s_{\text{h}}^{*}=\frac{\left[\pi_{\text{h}}\left(\rho +\mu_{\text{h}}\right)+\rho \tau i_{h}^{*}\right]\left[\mu_{\text{v}}\left(\sigma +\mu_{\text{v}}\right)\left(ap_{\text{v}}i_{\text{h}}^{*}+\mu_{\text{v}}\right)\right]}{\left[a^{2}\sigma p_{\text{h}}p_{\text{v}}\pi_{\text{v}}i_{\text{h}}^{*}+\mu_{\text{v}}\mu_{\text{h}}\left(\sigma +\mu_{\text{v}}\right)\left(ap_{\text{v}}i_{\text{h}}^{*}+\mu_{\text{v}}\right)\right]\left(\mu_{h}+\rho \right)},\\ e_{\text{h}}^{*}=\frac{\left(\omega +\tau +\mu_{\text{h}}\right)i_{\text{h}}^{*}}{\varepsilon },\\ r_{\text{h}}^{*}=\frac{\tau i_{\text{h}}^{*}}{\mu_{\text{h}}+\rho },\\ s_{\text{v}}^{*}=\frac{\pi_{\text{v}}}{ap_{\text{v}}i_{\text{h}}^{*}+\mu_{\text{v}}},\\ e_{\text{v}}^{*}=\frac{ap_{\text{v}}\pi_{\text{v}}i_{\text{h}}^{*}}{\left(\sigma +\mu_{\text{v}}\right)\left(ap_{\text{v}}i_{\text{h}}^{*}+\mu_{\text{v}}\right)},\\ i_{\text{v}}^{*}=\frac{a\sigma p_{\text{v}}\pi_{\text{v}}i_{\text{h}}^{*}}{\left(\sigma +\mu_{\text{v}}\right)\left(ap_{\text{v}}i_{\text{h}}^{*}+\mu_{\text{v}}\right)\mu_{\text{v}}},\\ i_{\text{h}}^{*}=\frac{\left(\rho +\mu_{\text{h}}\right)\left[a^{2}\varepsilon p_{\text{h}}p_{\text{v}}\pi_{\text{h}}\pi_{\text{v}}\sigma -\mu_{\text{v}}^{2}\mu_{\text{h}}\left(\varepsilon +\mu_{\text{h}}\right)\left(\sigma +\mu_{\text{v}}\right)\left(\mu_{\text{h}}+\tau +\omega \right)\right]}{B}, \end{array}\right. \end{array}$$

and the term

(13) $$\begin{array}{c} B=\left(ap_{\text{v}}\left(a\sigma p_{\text{h}}\pi_{\text{v}}\left(\varepsilon \rho \omega +\mu_{\text{h}}\left(\rho \left(\tau +\omega \right)+\varepsilon \left(\rho +\tau +\omega \right)+\mu_{\text{h}}\left(\varepsilon +\rho +\tau +\omega +\mu_{\text{h}}\right)\right)\right)+\mu_{\text{h}}\left(\varepsilon +\mu_{\text{h}}\right)\left(\rho +\mu_{\text{h}}\right)\left(\tau +\omega +\mu_{\text{h}}\right)\mu_{\text{v}}\left(\sigma +\mu_{\text{v}}\right)\right)\right). \end{array}$$

### 3.3. Basic Reproduction Number, *R*_0_

The basic reproduction number, *R*_0_, is defined as the number of secondary infections caused by one infected host or vector in a completely susceptible population [10]. The *next-generation matrix* approach as done by Van den Driessche and Watmough in [5, 11] is applied to derive

(14) $$\begin{array}{c} F=\left(\begin{array}{c} ap_{\text{h}}i_{\text{v}}s_{\text{h}}\\ 0\\ ap_{\text{v}}i_{\text{h}}s_{\text{v}} \end{array}\right),\\ V=\left(\begin{array}{c} \mu_{\text{h}}e_{\text{h}}+\varepsilon e_{\text{h}}\\ -\varepsilon e_{\text{h}}+\mu_{\text{h}}i_{\text{h}}+\omega i_{\text{h}}+\tau i_{\text{h}}\\ \mu_{\text{v}}e_{\text{v}}+\delta e_{\text{v}}\\ -\delta e_{\text{v}}+\mu_{\text{v}}i_{\text{v}} \end{array}\right). \end{array}$$

By denoting matrix *F*=(∂*𝔽*/∂*x*_*i*_) and *V*=(∂*𝕍*/∂*x*_*i*_), where *x*_*i*_=*e*_h_, *i*_h_, *e*_v_, *i*_v_, the spectral radius of the next-generation matrix *FV* ^−1^ gives the value of *R*_0_:

(15) $$\begin{array}{c} F=\left(\begin{array}{cccc} 0 & 0 & 0 & ap_{\text{h}}s_{\text{h}}\\ 0 & 0 & 0 & 0\\ 0 & ap_{\text{v}}s_{\text{v}} & 0 & 0\\ 0 & 0 & 0 & 0 \end{array}\right),\\ V=\left(\begin{array}{cccc} \varepsilon +\mu_{\text{h}} & 0 & 0 & 0\\ -\varepsilon & \mu_{\text{h}}+\omega +\tau & 0 & 0\\ 0 & 0 & \mu_{\text{v}}+\delta & 0\\ 0 & 0 & -\delta & \mu_{\text{v}} \end{array}\right),\\ FV^{-1}=\left(\begin{array}{cccc} 0 & 0 & \frac{a\delta p_{\text{h}}}{\mu_{\text{v}}\left(-\delta +\mu_{\text{v}}\right)} & \frac{-ap_{\text{h}}}{\mu_{\text{v}}}\\ 0 & \frac{ap_{\text{v}}}{\mu_{\text{h}}+\tau +\omega } & 0 & 0\\ 0 & 0 & 0 & 0\\ 0 & 0 & 0 & 0 \end{array}\right). \end{array}$$

The spectral radius *σ*(*FV* ^−1^) gives

(16) $$\begin{array}{c} R_{0}=\sigma \left(FV^{-1}\right)=\sqrt{\frac{a^{2}\varepsilon p_{\text{h}}p_{\text{v}}\pi_{\text{h}}\pi_{\text{v}}\sigma }{\mu_{\text{v}}^{2}\mu_{\text{h}}\left(\varepsilon +\mu_{\text{h}}\right)\left(\sigma +\mu_{\text{v}}\right)\left(\mu_{\text{h}}+\tau +\omega \right)}}. \end{array}$$

One infected human in a population of susceptible vectors will cause *R*_v_ infected vectors; likewise, one infected vector in a population will cause *R*_h_ infected humans [5]. Therefore, the basic reproduction number can be rewritten as $R_{0}=\sqrt{R_{\text{h}}R_{\text{v}}}$, where *R*_h_=(*aεp*_h_ *π*_h_/*μ*_h_(*μ*_h_+*ε*)(*μ*_h_+*τ*+*ω*)) and *R*_v_=(*σap*_v_ *π*_v_/*μ*_v_^2^(*σ*+*μ*_v_)). Thus, *R*_0_ can also be defined as the square root of the product of the number of infected humans in the susceptible population caused by one infected tsetse fly in its infectious lifetime and the number of infected tsetse flies caused by one infected human during the infectious period [12].

### 3.4. Local Stability of Disease-Free Equilibrium (DFE)

**Theorem 2 .**

If *R*_0_ ≤ 1, the DFE given by *E*_0_ is locally asymptotically stable in the region defined by (7), and it is unstable when *R*_0_ > 1.

*Proof*. The DFE is locally stable if all eigenvalues of Jacobian matrix *J*_*E*_0__ are negative. The matrix has all eigenvalues negative only if the trace of *J*_*E*_0__ < 0 and determinant of *J*_*E*_0__ > 0. By linearizing system (6) around *E*_0_, we obtain the following Jacobian matrix:

(17) $$\begin{array}{c} J_{E_{0}}=\left(\begin{array}{ccccccc} -\mu_{\text{h}} & 0 & 0 & \rho & 0 & 0 & -ap_{\text{h}}s_{\text{h}}\\ 0 & -\left(\varepsilon +\mu_{\text{h}}\right) & 0 & 0 & 0 & 0 & ap_{\text{h}}s_{\text{h}}\\ 0 & \varepsilon & -\left(\omega +\tau +\mu_{\text{h}}\right) & 0 & 0 & 0 & 0\\ 0 & 0 & \tau & -\left(\rho +\mu_{\text{h}}\right) & 0 & 0 & 0\\ 0 & 0 & -ap_{\text{v}}s_{\text{v}} & 0 & -\mu_{\text{v}} & 0 & 0\\ 0 & 0 & ap_{\text{v}}s_{\text{v}} & 0 & 0 & -\left(\sigma +\mu_{\text{v}}\right) & 0\\ 0 & 0 & 0 & 0 & 0 & \sigma & -\mu_{\text{v}} \end{array}\right). \end{array}$$

The trace of matrix *J*_*E*_0__ is such that

(18) $$\begin{array}{c} \text{tr}\left(J_{E_{0}}\right)=-\left(\mu_{\text{h}}+\varepsilon +\mu_{\text{h}}+\mu_{\text{h}}+\omega +\tau +\rho +\mu_{\text{h}}+\mu_{\text{v}}+\sigma +\mu_{\text{v}}+\mu_{\text{v}}\right)\\ =-\left(4\mu_{\text{h}}+3\mu_{\text{v}}+\varepsilon +\omega +\tau +\rho +\sigma \right)<0. \end{array}$$

Using the basic properties of matrix algebra as in [13], it is clear that the eigenvalues *λ*_1_=−*μ*_*h*_ and *λ*_2_=−*μ*_*v*_ of the matrix *J*_*E*_0__ have negative real parts. The reduced matrix is

(19) $$\begin{array}{c} J_{E_{1}}=\left(\begin{array}{ccccc} -\left(\varepsilon +\mu_{\text{h}}\right) & 0 & 0 & 0 & ap_{\text{h}}s_{\text{h}}\\ \varepsilon & -\left(\omega +\tau +\mu_{\text{h}}\right) & 0 & 0 & 0\\ 0 & \tau & -\left(\rho +\mu_{\text{h}}\right) & 0 & 0\\ 0 & ap_{\text{v}}s_{\text{v}} & 0 & -\left(\sigma +\mu_{\text{v}}\right) & 0\\ 0 & 0 & 0 & \sigma & -\mu_{\text{v}} \end{array}\right). \end{array}$$

From matrix *J*_*E*_1__, the eigenvalue *λ*_3_=−(*ρ*+*μ*_h_) has negative real part. The remaining matrix is further reduced by using the reduction techniques, and we obtain

(20) $$\begin{array}{c} J_{E_{2}}=\left(\begin{array}{cccc} -\left(\varepsilon +\mu_{\text{h}}\right) & 0 & 0 & ap_{\text{h}}s_{\text{h}}\\ 0 & -\left(\omega +\tau +\mu_{\text{h}}\right) & 0 & \frac{ap_{\text{h}}s_{\text{h}}\varepsilon }{\varepsilon +\mu_{\text{h}}}\\ 0 & ap_{\text{v}}s_{\text{v}} & -\left(\sigma +\mu_{\text{v}}\right) & 0\\ 0 & 0 & \sigma & -\mu_{\text{v}} \end{array}\right). \end{array}$$

Using the properties of matrix algebra, the matrix *J*_*E*_2__ has eigenvalue −(*ε*+*μ*_h_) which has negative real part. We further reduce to a 2 × 2 matrix by using the same reduction techniques. The matrix is

(21) $$\begin{array}{c} J_{E_{3}}=\left(\begin{array}{cc} -\left(\omega +\tau +\mu_{\text{h}}\right) & \frac{ap_{\text{h}}s_{\text{h}}\varepsilon }{\varepsilon +\mu_{\text{h}}}\\ \frac{ap_{\text{v}}s_{\text{v}}\sigma }{\sigma +\mu_{\text{v}}} & -\mu_{\text{v}} \end{array}\right). \end{array}$$

From the reduced 2 × 2 matrix, the trace is negative and the determinant is

(22) $$\begin{array}{c} \text{Det}\left(J_{E_{3}}\right)=\left(\omega +\tau +\mu_{\text{h}}\right)\mu_{\text{v}}-\frac{ap_{\text{h}}s_{\text{h}}\varepsilon }{\varepsilon +\mu_{\text{h}}}\times \frac{ap_{\text{v}}s_{\text{v}}\sigma }{\sigma +\mu_{\text{v}}}\\ =\left(\omega +\tau +\mu_{\text{h}}\right)\mu_{\text{v}}\left[1-\frac{a^{2}\varepsilon p_{\text{h}}p_{\text{v}}\pi_{\text{h}}\pi_{\text{v}}\sigma }{\mu_{\text{v}}^{2}\mu_{\text{h}}\left(\varepsilon +\mu_{\text{h}}\right)\left(\sigma +\mu_{\text{v}}\right)\left(\mu_{\text{h}}+\tau +\omega \right)}\right]. \end{array}$$

Since

(23) $$\begin{array}{c} R_{0}=\sqrt{\frac{a^{2}\varepsilon p_{\text{h}}p_{\text{v}}\pi_{\text{h}}\pi_{\text{v}}\sigma }{\mu_{\text{v}}^{2}\mu_{\text{h}}\left(\varepsilon +\mu_{\text{h}}\right)\left(\sigma +\mu_{\text{v}}\right)\left(\mu_{\text{h}}+\tau +\omega \right)}}, \end{array}$$

then, by letting *R*_*T*_=(*a* ^2^ *εp*_h_ *p*_v_ *π*_h_ *π*_v_ *σ*/*μ*_v_^2^ *μ*_h_(*ε*+*μ*_h_)(*σ*+*μ*_v_)(*μ*_h_+*τ*+*ω*)), we find our determinant as

(24) $$\begin{array}{c} \text{Det}\left(J_{E_{3}}\right)=\left(\omega +\tau +\mu_{\text{h}}\right)\mu_{\text{v}}\left[1-R_{\text{T}}\right]. \end{array}$$

The value of *R*_T_ can be seen to be positive because all the parameters are positive. As a result, the determinant in (24) is positive if and only if *R*_T_ < 1. Therefore, the DFE is locally stable if *R*_T_ ≤ 1.

### 3.5. Global Stability of Disease-Free Equilibrium (DFE)

To show that the DFE is globally stable, we apply Lyapunov's theorem in [5].

**Theorem 3 .**

The DFE defined by *E*_0_ is globally asymptotically stable in the region defined by (7) if *R*_0_ ≤ 1. Otherwise unstable if *R*_0_ > 1.

*Proof*. We define Lyapunov's function as

(25) $$\begin{array}{c} V=k_{1}\left(s_{\text{h}}-s_{{\text{h}}_{0}}-s_{{\text{h}}_{0}}\ln\frac{s_{\text{h}}}{s_{{\text{h}}_{0}}}\right)+k_{2}e_{\text{h}}+k_{3}i_{\text{h}}+k_{4}\left(s_{\text{v}}-s_{{\text{v}}_{0}}-s_{{\text{v}}_{0}}\ln\frac{s_{\text{v}}}{s_{{\text{v}}_{0}}}\right)+k_{5}e_{\text{v}}+k_{6}i_{\text{v}}, \end{array}$$

satisfying system (6), where *k*_1_, *k*_2_, *k*_3_, *k*_4_, *k*_5_, *k*_6_ > 0 are to be determined and *s*_h_0__=(*π*_*h*_/*μ*_*h*_) and *s*_v_0__=(*π*_v_/*μ*_v_). We first show that *V* > 0 for all *E* ≠ ((*π*_h_/*μ*_h_), 0, 0, 0, (*π*_v_/*μ*_v_), 0, 0). It is enough to check that

(26) $$\begin{array}{c} k_{1}s_{{\text{h}}_{0}}\left(\frac{s_{\text{h}}}{s_{{\text{h}}_{0}}}-1-\ln\frac{s_{\text{h}}}{s_{{\text{h}}_{0}}}\right)>0,\\ k_{4}s_{{\text{v}}_{0}}\left(\frac{s_{\text{v}}}{s_{{\text{v}}_{0}}}-1-\ln\frac{s_{\text{v}}}{s_{{\text{v}}_{0}}}\right)>0. \end{array}$$

The function *g*(*m*)=*m* − 1 − ln*m* such that *m*=(*s*_h_/*s*_h_0__)=(*s*_v_/*s*_v_0__) has minimum value equal to zero when *m*=1; hence, *g*(*m*) > 0 for all *m* > 0. Thus, Lyapunov's function *V* > 0. The function *V* is radially unbounded because as |*m*|⟶*∞*, the function *g*(*m*)⟶*∞*. We now take the derivative of *V* with respect to time and use system (6) to replace the derivatives in the right hand side such that

(27) $$\begin{array}{c} V^{\prime }=k_{1}\left(1-\frac{s_{{\text{h}}_{0}}}{s_{\text{h}}}\right)\frac{ds_{\text{h}}}{dt}+k_{2}\frac{de_{\text{h}}}{dt}+k_{3}\frac{di_{\text{h}}}{dt}+k_{4}\left(1-\frac{s_{{\text{v}}_{0}}}{s_{\text{v}}}\right)\frac{ds_{\text{v}}}{dt}+k_{5}\frac{de_{\text{v}}}{dt}+k_{6}\frac{di_{\text{v}}}{dt},\\ =k_{1}\left(1-\frac{s_{{\text{h}}_{0}}}{s_{\text{h}}}\right)\left[\pi_{\text{h}}+\rho r_{\text{h}}-ap_{\text{h}}i_{\text{v}}s_{\text{h}}-\mu_{\text{h}}s_{\text{h}}\right]+k_{2}\left[ap_{\text{h}}i_{\text{v}}s_{\text{h}}-\varepsilon e_{\text{h}}-\mu_{\text{h}}e_{\text{h}}\right]+k_{3}\left[\varepsilon e_{\text{h}}-\mu_{\text{h}}i_{\text{h}}-\omega i_{\text{h}}-\tau i_{\text{h}}\right]+k_{4}\left(1-\frac{s_{{\text{v}}_{0}}}{s_{\text{v}}}\right)\left[\pi_{\text{v}}-\mu_{\text{v}}s_{\text{v}}-ap_{\text{v}}i_{\text{h}}s_{\text{v}}\right]+k_{5}\left[ap_{\text{v}}i_{\text{h}}s_{\text{v}}-\mu_{\text{v}}e_{\text{v}}-\sigma e_{\text{v}}\right]+k_{6}\left[\sigma e_{\text{v}}-\mu_{\text{v}}i_{\text{v}}\right],\\ =2k_{1}\pi_{\text{h}}-ap_{\text{h}}s_{\text{h}}k_{1}i_{\text{v}}+\rho r_{\text{h}}k_{1}-k_{1}\mu_{\text{h}}s_{\text{h}}-k_{1}\frac{\pi_{\text{h}}^{2}}{\mu_{\text{h}}s_{\text{h}}}+\frac{ap_{\text{h}}\pi_{\text{h}}i_{\text{v}}}{\mu_{\text{h}}}k_{1}-\frac{\pi_{\text{h}}\rho r_{\text{h}}}{\mu_{\text{h}}s_{\text{h}}}k_{1}+k_{2}ap_{\text{h}}s_{\text{h}}i_{\text{v}}-k_{2}\left(\varepsilon +\mu_{\text{h}}\right)e_{\text{h}}+k_{3}\varepsilon e_{\text{h}}-k_{3}\left(\tau +\mu_{\text{h}}+\omega \right)i_{\text{h}}+2k_{4}\pi_{\text{v}}-k_{4}ap_{\text{v}}s_{\text{v}}i_{\text{h}}-\mu_{\text{v}}s_{\text{v}}k_{4}-\frac{\pi_{\text{v}}^{2}}{\mu_{\text{v}}s_{\text{v}}}k_{4}+\frac{ap_{\text{v}}i_{\text{h}}\pi_{\text{v}}}{\mu_{\text{v}}}k_{4}-k_{5}\left(\mu_{\text{v}}+\sigma \right)e_{\text{v}}+k_{5}ap_{\text{v}}s_{\text{v}}i_{\text{h}}+k_{6}\sigma e_{\text{v}}-k_{6}\mu_{\text{v}}i_{\text{v}}. \end{array}$$

The terms with *r*_h_ are ignored because if *s*_h_, *e*_h_, *i*_h_ are globally stable, then *r*_h_⟶0 at any time *t* and the DFE for system (6) is globally stable. Taking *k*_1_=*k*_2_=(1/*μ*_h_+*ε*), *k*_4_=*k*_5_=(1/*μ*_v_+*σ*), *k*_3_=(1/*ε*), and *k*_6_=(1/*σ*), the derivative of *V* with respect to time becomes

(28) $$\begin{array}{c} V^{\prime }=-\frac{\pi_{\text{h}}}{\mu_{\text{h}}+\varepsilon }\left(\frac{\pi_{\text{h}}}{\mu_{\text{h}}s_{\text{h}}}+\frac{\mu_{\text{h}}s_{\text{h}}}{\pi_{\text{h}}}-2\right)-\frac{\left(\tau +\mu_{\text{h}}+\omega \right)}{\varepsilon }i_{\text{h}}+\frac{ap_{\text{h}}\pi_{\text{h}}}{\left(\mu_{\text{h}}+\varepsilon \right)\mu_{\text{h}}}i_{\text{v}}-\frac{\pi_{\text{v}}}{\mu_{\text{v}}+\sigma }\left(\frac{\pi_{\text{v}}}{\mu_{\text{v}}s_{\text{v}}}+\frac{\mu_{\text{v}}s_{\text{v}}}{\pi_{\text{v}}}-2\right)-\frac{\mu_{\text{v}}}{\sigma }i_{\text{v}}+\frac{ap_{\text{v}}\pi_{\text{v}}}{\mu_{\text{v}}\left(\mu_{\text{v}}+\sigma \right)}i_{\text{h}}\\ =-\left[\frac{\pi_{\text{h}}}{\mu_{\text{h}}+\varepsilon }\left(\frac{\pi_{\text{h}}}{\mu_{\text{h}}s_{\text{h}}}+\frac{\mu_{\text{h}}s_{\text{h}}}{\pi_{\text{h}}}-2\right)+\frac{\pi_{\text{v}}}{\mu_{\text{v}}+\sigma }\left(\frac{\pi_{\text{v}}}{\mu_{\text{v}}s_{\text{v}}}+\frac{\mu_{\text{v}}s_{\text{v}}}{\pi_{\text{v}}}-2\right)\right]+\frac{\left(\tau +\mu_{\text{h}}+\omega \right)}{\varepsilon }\left(\frac{\varepsilon ap_{\text{v}}\pi_{\text{v}}}{\mu_{\text{v}}\left(\sigma +\mu_{\text{v}}\right)\left(\mu_{\text{h}}+\tau +\omega \right)}-1\right)i_{\text{h}}+\frac{\mu_{\text{v}}}{\sigma }\left(\frac{a\sigma p_{\text{h}}\pi_{\text{h}}}{\mu_{\text{v}}\mu_{\text{h}}\left(\mu_{\text{h}}+\varepsilon \right)}-1\right)i_{\text{v}}\\ =-\left[\frac{\pi_{\text{h}}}{\mu_{\text{h}}+\varepsilon }\left(\frac{\pi_{\text{h}}}{\mu_{\text{h}}s_{\text{h}}}+\frac{\mu_{\text{h}}s_{\text{h}}}{\pi_{\text{h}}}-2\right)+\frac{\pi_{\text{v}}}{\mu_{\text{v}}+\sigma }\left(\frac{\pi_{\text{v}}}{\mu_{\text{v}}s_{\text{v}}}+\frac{\mu_{\text{v}}s_{\text{v}}}{\pi_{\text{v}}}-2\right)\right]+\frac{\left(\tau +\mu_{\text{h}}+\omega \right)}{\varepsilon }\left(R_{\text{v}}-1\right)i_{\text{h}}+\frac{\mu_{v}}{\sigma }\left(R_{\text{h}}-1\right)i_{\text{v}}. \end{array}$$

The terms ((*π*_h_/*μ*_h_ *s*_h_)+(*μ*_h_ *s*_h_/*π*_h_) − 2) and ((*π*_v_/*μ*_v_ *s*_v_)+(*μ*_v_ *s*_v_/*π*_v_) − 2) are positive because if we suppose *m*=(*π*_h_/*μ*_h_ *s*_h_)=(*π*_v_/*μ*_v_ *s*_v_), we have *m*+(1/*m*) − 2=(*m* ^2^ − 2*m*+1/*m*)=((*m* − 1)^2^/*m*) > 0 for all *m* > 1 and since *R*_v_ ≤ 1 and *R*_h_ ≤ 1, then *V*′ is negative. Thus, we have *V*′ < 0 for all *E*_0_ ≠ ((*π*_h_/*μ*_h_), 0, 0, 0, (*π*_v_/*μ*_v_), 0, 0).

Thus, the largest compact invariant set in *D* is the singleton set *E*_0_. Hence, system (6) is globally asymptotically stable.

### 3.6. Local Stability of Endemic Equilibrium (EE)

**Theorem 4 .**

The unique endemic equilibrium defined by *E* ^*∗*^ is locally asymptotically stable in the region defined by (7) if *R*_0_ > 1, but is unstable if *R*_0_ ≤ 1.

*Proof*. We give the proof of this theorem based on the approach used by Olaniyi and Obabiyi [9, 14]. From the *EE* points defined in (12), since all values are positive, we express the value of *i*_h_^*∗*^ in terms of *R*_0_ to obtain

(29) $$\begin{array}{c} i_{\text{h}}^{*}=\frac{\mu_{\text{v}}^{2}\mu_{\text{h}}\left(\varepsilon +\mu_{\text{h}}\right)\left(\rho +\mu_{\text{h}}\right)\left(\sigma +\mu_{\text{v}}\right)\left(\mu_{\text{h}}+\tau +\omega \right)}{B}\left[\frac{a^{2}\varepsilon p_{\text{h}}p_{\text{v}}\pi_{\text{h}}\pi_{\text{v}}\sigma }{\mu_{\text{v}}^{2}\mu_{\text{h}}\left(\varepsilon +\mu_{\text{h}}\right)\left(\sigma +\mu_{\text{v}}\right)\left(\mu_{\text{h}}+\tau +\omega \right)}-1\right]\\ =\frac{\mu_{\text{v}}^{2}\mu_{\text{h}}\left(\varepsilon +\mu_{\text{h}}\right)\left(\rho +\mu_{\text{h}}\right)\left(\sigma +\mu_{\text{v}}\right)\left(\mu_{\text{h}}+\tau +\omega \right)}{B}\left[R_{0}^{2}-1\right], \end{array}$$

where

(30) $$\begin{array}{c} B=\left(ap_{\text{v}}\left(a\sigma p_{\text{h}}\pi_{\text{v}}\left(\varepsilon \rho \omega +\mu_{\text{h}}\left(\rho \left(\tau +\omega \right)+\varepsilon \left(\rho +\tau +\omega \right)+\mu_{\text{h}}\left(\varepsilon +\rho +\tau +\omega +\mu_{\text{h}}\right)\right)\right)+\mu_{\text{h}}\left(\varepsilon +\mu_{\text{h}}\right)\left(\rho +\mu_{\text{h}}\right)\left(\tau +\omega +\mu_{\text{h}}\right)\mu_{\text{v}}\left(\sigma +\mu_{\text{v}}\right)\right)\right). \end{array}$$

Since the basic reproduction number $R_{0}=\sqrt{\left(a^{2}\varepsilon p_{\text{h}}p_{\text{v}}\pi_{\text{h}}\pi_{\text{v}}\sigma /\mu_{\text{v}}^{2}\mu_{\text{h}}\left(\varepsilon +\mu_{\text{h}}\right)\left(\sigma +\mu_{\text{v}}\right)\left(\mu_{h}+\tau +\omega \right)\right)}$, if we let

(31) $$\begin{array}{c} R_{\text{T}}=\frac{a^{2}\varepsilon p_{\text{h}}p_{\text{v}}\pi_{\text{h}}\pi_{\text{v}}\sigma }{\mu_{\text{v}}^{2}\mu_{\text{h}}\left(\varepsilon +\mu_{\text{h}}\right)\left(\sigma +\mu_{\text{v}}\right)\left(\mu_{\text{h}}+\tau +\omega \right)}, \end{array}$$

then we can find *i*_*h*_^*∗*^ as

(32) $$\begin{array}{c} i_{\text{h}}^{*}=\frac{\mu_{\text{v}}^{2}\mu_{\text{h}}\left(\varepsilon +\mu_{\text{h}}\right)\left(\rho +\mu_{\text{h}}\right)\left(\sigma +\mu_{\text{v}}\right)\left(\mu_{\text{h}}+\tau +\omega \right)}{B}\left[R_{\text{T}}-1\right]. \end{array}$$

The value of *B* is clearly positive because all parameters are positive. Hence, *i*_h_^*∗*^ > 0 if and only if *R*_T_ > 1, implying that the *EE* is locally asymptotically stable if *R*_T_ > 1.

### 3.7. Global Stability of Endemic Equilibrium (EE)

To show the global stability of the *EE*, we use Lyapunov's theorem together with the following lemma.

**Lemma 1 .**

Suppose that *y*_1_, *y*_2_, ⋯, *y*_*n*_ are *n* positive numbers. Then, their arithmetic mean is greater than or equal to the geometric mean, that is (*y*_1_+*y*_2_+⋯+*y*_*n*_/*n*) ≥ (*y*_1_ *y*_2_ ⋯ *y*_*n*_)^1/*n*^.

**Theorem 5 .**

The EE defined by *E* ^*∗*^ is globally asymptotically stable if *R*_0_ > 1, otherwise unstable.

*Proof*. The proof is based on the idea as explained by Martcheva [5]. We define Lyapunov's function as

(33) $$\begin{array}{c} V=k_{1}\left(s_{\text{h}}-s_{\text{h}}^{*}-s_{\text{h}}^{*}\ln\frac{s_{\text{h}}}{s_{\text{h}}^{*}}\right)+k_{2}\left(e_{\text{h}}-e_{\text{h}}^{*}-e_{\text{h}}^{*}\ln\frac{e_{\text{h}}}{e_{\text{h}}^{*}}\right)+k_{3}\left(i_{\text{h}}-i_{\text{h}}^{*}-i_{\text{h}}^{*}\ln\frac{i_{\text{h}}}{i_{\text{h}}^{*}}\right)+k_{4}\left(s_{\text{v}}-s_{\text{v}}^{*}-s_{\text{v}}^{*}\ln\frac{s_{\text{v}}}{s_{\text{v}}^{*}}\right)+k_{5}\left(e_{\text{v}}-e_{\text{v}}^{*}-e_{\text{v}}^{*}\ln\frac{e_{\text{v}}}{e_{\text{v}}^{*}}\right)+k_{6}\left(i_{\text{v}}-i_{\text{v}}^{*}-i_{\text{v}}^{*}\ln\frac{i_{\text{v}}}{i_{\text{v}}^{*}}\right), \end{array}$$

satisfying system (6) with *k*_1_, *k*_2_, *k*_3_, *k*_4_, *k*_5_, *k*_6_ > 0 to be determined. The function V is nonnegative for all (*s*_h_, *e*_h_, *i*_h_, *r*_h_, *s*_v_, *e*_v_, *i*_v_) ≠ (*s*_h_^*∗*^, *e*_h_^*∗*^, *i*_h_^*∗*^, *r*_h_^*∗*^, *s*_v_^*∗*^, *e*_v_^*∗*^, *i*_v_^*∗*^) and radially unbounded.

We need to prove that *V*′ < 0 for all (*s*_h_, *e*_h_, *i*_h_, *r*_h_, *s*_v_, *e*_v_, *i*_v_) ≠ (*s*_h_^*∗*^, *e*_h_^*∗*^, *i*_h_^*∗*^, *r*_h_^*∗*^, *s*_v_^*∗*^, *e*_v_^*∗*^, *i*_v_^*∗*^). We find the derivative of *V* with respect to time and replace the derivatives *s*_*h*_′, *e*_*h*_′, *i*_*h*_′, *r*_*h*_′, *s*_*v*_′, *e*_*v*_′, *i*_*v*_′ with system (6). We also ignore the *r*_*h*_ terms because if *s*_*h*_, *e*_*h*_, *i*_*h*_ are globally stable, then *r*_*h*_⟶0 at any time *t* and *EE* is globally stable:

(34) $$\begin{array}{c} V^{\prime }=k_{1}\left(1-\frac{s_{\text{h}}^{*}}{s_{\text{h}}}\right)\left[\pi_{\text{h}}-ap_{\text{h}}i_{\text{v}}s_{\text{h}}-\mu_{\text{h}}s_{\text{h}}\right]+k_{2}\left(1-\frac{e_{\text{h}}^{*}}{e_{\text{h}}}\right)\left[ap_{\text{h}}i_{\text{v}}s_{\text{h}}-\left(\mu_{\text{h}}+\varepsilon \right)e_{\text{h}}\right]+k_{3}\left(1-\frac{i_{\text{h}}^{*}}{i_{\text{h}}}\right)\left[\varepsilon e_{\text{h}}-\left(\omega +\tau +u_{\text{h}}\right)i_{\text{h}}\right]+k_{4}\left(1-\frac{s_{\text{v}}^{*}}{s_{\text{v}}}\right)\left[\pi_{\text{v}}-ap_{\text{v}}i_{\text{h}}s_{\text{v}}-\mu_{\text{v}}s_{\text{v}}\right]+k_{5}\left(1-\frac{e_{\text{v}}^{*}}{e_{\text{v}}}\right)\left[ap_{\text{v}}i_{\text{h}}s_{\text{v}}-\left(\mu_{\text{v}}+\sigma \right)e_{\text{v}}\right]+k_{6}\left(1-\frac{i_{\text{v}}^{*}}{i_{\text{v}}}\right)\left[\sigma e_{\text{v}}-u_{\text{v}}i_{\text{v}}\right]. \end{array}$$

We now substitute *π*_*h*_=*ap*_*h*_ *i*_*v*_^*∗*^ *s*_*h*_^*∗*^+*μ*_*h*_ *s*_*h*_^*∗*^ and *π*_*v*_=*ap*_*v*_ *i*_*h*_^*∗*^ *s*_*v*_^*∗*^+*μ*_*v*_ *s*_*v*_^*∗*^ at the endemic equilibrium and then simplify and put similar terms together to obtain

(35) $$\begin{array}{c} V^{\prime }=-k_{1}\frac{{\left(s_{\text{h}}-s_{\text{h}}^{*}\right)}^{2}\mu_{\text{h}}}{s_{\text{h}}}+k_{1}ap_{\text{h}}i_{\text{v}}^{*}s_{\text{h}}^{*}-k_{1}ap_{\text{h}}i_{\text{v}}s_{\text{h}}-k_{1}ap_{\text{h}}\frac{s_{\text{h}}^{*2}i_{\text{v}}^{*}}{s_{\text{h}}}+k_{1}ap_{\text{h}}i_{\text{v}}s_{\text{h}}^{*}+k_{2}ap_{\text{h}}s_{\text{h}}i_{\text{v}}-k_{2}\left(\varepsilon +\mu_{\text{h}}\right)e_{\text{h}}-k_{2}ap_{\text{h}}s_{\text{h}}i_{\text{v}}\frac{e_{\text{h}}^{*}}{e_{\text{h}}}+k_{2}\left(\varepsilon +\mu_{\text{h}}\right)e_{\text{h}}^{*}+k_{3}\varepsilon e_{\text{h}}-k_{3}\left(\omega +\tau +\mu_{\text{h}}\right)i_{\text{h}}^{*}-k_{3}\varepsilon e_{\text{h}}\frac{i_{\text{h}}^{*}}{i_{\text{h}}}+k_{3}\left(\omega +\tau +\mu_{\text{h}}\right)i_{\text{h}}^{*}-k_{4}\frac{{\left(s_{\text{v}}-s_{\text{v}}^{*}\right)}^{2}\mu_{\text{v}}}{s_{\text{v}}}+k_{4}ap_{\text{v}}i_{\text{h}}^{*}s_{\text{v}}^{*}-k_{4}ap_{\text{v}}i_{\text{h}}s_{\text{v}}-k_{4}ap_{\text{v}}\frac{s_{\text{v}}^{*2}i_{\text{h}}^{*}}{s_{\text{v}}}+k_{4}ap_{\text{v}}i_{\text{h}}s_{\text{v}}^{*}+k_{5}ap_{\text{v}}s_{\text{v}}i_{\text{h}}-k_{5}\left(\sigma +\mu_{\text{v}}\right)e_{\text{v}}-k_{5}ap_{\text{v}}s_{\text{v}}i_{\text{h}}\frac{e_{\text{v}}^{*}}{e_{\text{v}}}+k_{5}\left(\sigma +\mu_{\text{v}}\right)e_{\text{v}}^{*}+k_{6}\sigma e_{\text{v}}-k_{6}\mu_{\text{v}}i_{\text{v}}^{*}-k_{6}\sigma e_{\text{v}}\frac{i_{\text{v}}^{*}}{i_{\text{v}}}+k_{6}\mu_{\text{v}}i_{\text{v}}^{*}. \end{array}$$

We suppose *k*_1_=*k*_2_ and *k*_4_=*k*_5_ and multiply and divide the same equilibrium value to some of the fractions to obtain

(36) $$\begin{array}{c} V^{\prime }=-k_{1}\frac{{\left(s_{\text{h}}-s_{\text{h}}^{*}\right)}^{2}\mu_{\text{h}}}{s_{\text{h}}}+k_{1}ap_{\text{h}}i_{\text{v}}^{*}s_{\text{h}}^{*}-k_{1}ap_{\text{h}}\frac{s_{\text{h}}^{*2}i_{\text{v}}^{*}}{s_{\text{h}}}+k_{1}ap_{\text{h}}i_{\text{v}}s_{\text{h}}^{*}-k_{2}\left(\varepsilon +\mu_{\text{h}}\right)e_{\text{h}}-k_{2}ap_{\text{h}}s_{\text{h}}i_{\text{v}}\frac{e_{\text{h}}^{*}s_{\text{h}}^{*}i_{\text{v}}^{*}}{s_{\text{h}}^{*}i_{\text{v}}^{*}e_{\text{h}}}+k_{2}\left(\varepsilon +\mu_{\text{h}}\right)e_{\text{h}}^{*}+k_{3}\varepsilon e_{\text{h}}-k_{3}\left(\omega +\tau +\mu_{\text{h}}\right)i_{\text{h}}-k_{3}\varepsilon e_{\text{h}}\frac{i_{\text{h}}^{*}e_{\text{h}}^{*}}{i_{\text{h}}e_{\text{h}}^{*}}+k_{3}\left(\omega +\tau +\mu_{\text{h}}\right)i_{\text{h}}^{*}-k_{4}\frac{{\left(s_{\text{v}}-s_{\text{v}}^{*}\right)}^{2}\mu_{\text{v}}}{s_{\text{v}}}+k_{4}ap_{\text{v}}i_{\text{h}}^{*}s_{\text{v}}^{*}-k_{4}ap_{\text{v}}\frac{s_{\text{v}}^{*2}i_{\text{h}}^{*}}{s_{\text{v}}}+k_{4}ap_{\text{v}}i_{\text{h}}s_{\text{v}}^{*}-k_{5}\left(\sigma +\mu_{\text{v}}\right)e_{\text{v}}-k_{5}ap_{\text{v}}s_{\text{v}}i_{\text{h}}\frac{e_{\text{v}}^{*}s_{\text{v}}^{*}i_{\text{h}}^{*}}{s_{\text{v}}^{*}i_{\text{h}}^{*}e_{\text{v}}}+k_{5}\left(\sigma +\mu_{\text{v}}\right)e_{\text{v}}^{*}+k_{6}\sigma e_{\text{v}}-k_{6}\mu_{\text{v}}i_{\text{v}}^{*}-k_{6}\sigma e_{\text{v}}\frac{e_{\text{v}}^{*}i_{\text{v}}^{*}}{i_{\text{v}}e_{\text{v}}^{*}}+k_{6}\mu_{\text{v}}i_{\text{v}}^{*}. \end{array}$$

We choose *k*_3_=*k*_2_(*μ*_h_+*ε*/*ε*) such that *k*_3_(*ω*+*τ*+*μ*_h_)*i*_h_^*∗*^=*k*_2_(*ε*+*μ*_h_)*e*_h_^*∗*^ and choose *k*_6_=*k*_5_(*μ*_h_+*σ*/*σ*) such that *k*_6_ *μ*_v_ *i*_v_^*∗*^=*k*_5_(*σ*+*μ*_v_)*e*_v_^*∗*^. Now, *ap*_h_ *s*_h_^*∗*^ *i*_v_^*∗*^=(*μ*_h_+*ε*)*e*_h_^*∗*^ and *ap*_v_ *s*_v_^*∗*^ *i*_v_^*∗*^=(*μ*_v_+*σ*)*e*_v_^*∗*^ because *k*_1_=*k*_2_ and *k*_4_=*k*_5_, respectively. We now obtain

(37) $$\begin{array}{c} V^{\prime }=-k_{1}\frac{{\left(s_{\text{h}}-s_{\text{h}}^{*}\right)}^{2}\mu_{\text{h}}}{s_{\text{h}}}+k_{1}ap_{\text{h}}i_{\text{v}}^{*}s_{\text{h}}^{*}\left[3-\frac{s_{\text{h}}^{*}}{s_{\text{h}}}-\frac{e_{\text{h}}^{*}i_{\text{v}}s_{\text{h}}}{s_{\text{h}}^{*}e_{\text{h}}i_{\text{v}}^{*}}-\frac{e_{h}i_{h}^{*}}{i_{\text{h}}e_{\text{h}}^{*}}\right]+k_{1}ap_{\text{h}}i_{\text{v}}s_{\text{h}}^{*}-k_{3}\left(\omega +\tau +\mu_{\text{h}}\right)i_{\text{h}}+\left[k_{3}\varepsilon -k_{2}\left(\varepsilon +\mu_{\text{h}}\right)\right]e_{\text{h}}-k_{4}\frac{{\left(s_{\text{v}}-s_{\text{v}}^{*}\right)}^{2}\mu_{\text{v}}}{s_{\text{v}}}+k_{4}ap_{\text{v}}i_{\text{h}}^{*}s_{\text{v}}^{*}\left[3-\frac{s_{\text{v}}^{*}}{s_{\text{v}}}-\frac{e_{\text{v}}^{*}i_{\text{h}}s_{\text{v}}}{s_{\text{v}}^{*}e_{\text{v}}i_{\text{h}}^{*}}-\frac{e_{\text{v}}i_{\text{v}}^{*}}{i_{\text{v}}e_{\text{v}}^{*}}\right]+k_{4}ap_{\text{v}}i_{\text{h}}s_{\text{v}}^{*}-k_{6}\mu_{\text{v}}i_{\text{v}}+\left[k_{6}\sigma -k_{5}\left(\sigma +\mu_{\text{v}}\right)\right]e_{\text{v}}. \end{array}$$

Suppose *k*_6_=(*k*_1_ *ap*_h_ *s*_h_^*∗*^/*μ*_v_) and *k*_3_=(*k*_4_ *ap*_v_ *s*_v_^*∗*^/*ω*+*τ*+*μ*_h_). We then substitute and simplify to get

(38) $$\begin{array}{c} V^{\prime }=-k_{1}\frac{{\left(s_{\text{h}}-s_{\text{h}}^{*}\right)}^{2}\mu_{\text{h}}}{s_{\text{h}}}+k_{1}ap_{\text{h}}i_{\text{v}}^{*}s_{\text{h}}^{*}\left[3-\frac{s_{\text{h}}^{*}}{s_{\text{h}}}-\frac{e_{\text{h}}^{*}i_{\text{v}}s_{\text{h}}}{s_{\text{h}}^{*}e_{\text{h}}i_{\text{v}}^{*}}-\frac{e_{\text{h}}i_{\text{h}}^{*}}{i_{\text{h}}e_{\text{h}}^{*}}\right]-k_{4}\frac{{\left(s_{\text{v}}-s_{\text{v}}^{*}\right)}^{2}\mu_{\text{v}}}{s_{\text{v}}}+k_{4}ap_{\text{v}}i_{\text{h}}^{*}s_{\text{v}}^{*}\left[3-\frac{s_{\text{v}}^{*}}{s_{\text{v}}}-\frac{e_{\text{v}}^{*}i_{\text{h}}s_{\text{v}}}{s_{\text{v}}^{*}e_{\text{v}}i_{\text{h}}^{*}}-\frac{e_{\text{v}}i_{\text{v}}^{*}}{i_{\text{v}}e_{\text{v}}^{*}}\right]. \end{array}$$

From Lemma 1, the terms

(39) $$\begin{array}{c} k_{1}ap_{\text{h}}i_{\text{v}}^{*}s_{\text{h}}^{*}\left[3-\frac{s_{\text{h}}^{*}}{s_{\text{h}}}-\frac{e_{\text{h}}^{*}i_{\text{v}}s_{\text{h}}}{s_{\text{h}}^{*}e_{\text{h}}i_{\text{v}}^{*}}-\frac{e_{\text{h}}i_{\text{h}}^{*}}{i_{\text{h}}e_{\text{h}}^{*}}\right],\\ k_{4}ap_{\text{v}}i_{\text{h}}^{*}s_{\text{v}}^{*}\left[3-\frac{s_{\text{v}}^{*}}{s_{\text{v}}}-\frac{e_{\text{v}}^{*}i_{\text{h}}s_{\text{v}}}{s_{\text{v}}^{*}e_{\text{v}}i_{\text{h}}^{*}}-\frac{e_{\text{v}}i_{\text{v}}^{*}}{i_{\text{v}}e_{\text{v}}^{*}}\right]\leq 0. \end{array}$$

Therefore, *V*′ < 0 for all (*s*_h_, *e*_h_, *i*_h_, *r*_h_, *s*_v_, *e*_v_, *i*_v_) ≠ (*s*_h_^*∗*^, *e*_h_^*∗*^, *i*_h_^*∗*^, *r*_h_^*∗*^, *s*_v_^*∗*^, *e*_v_^*∗*^, *i*_v_^*∗*^), implying that the endemic equilibrium is globally asymptotically stable if *R*_0_ > 1.

## 4. Analysis of Optimal Control Model

In this section, we formulate the optimal control model by modifying system (6) to an optimal control problem. We thus define some linear functions *c*_*i*_(*t*)=1, for *i*=1, 2, 3. It is important to note that controls are fully effective when *c*_*i*_(*t*)=1 and not effective when *c*_*i*_(*t*)=0. The forces of infection *ξ*_h_ and *xi*_*v*_, which correspond to the human and vector population, respectively, are reduced by the factor (1 − *c*_1_), where *c*_1_ measures the level of success obtained due to the effort of educating people on the dangers of exposing their skin, and encouraging them to wear long sleeves and long pants during the day to minimize tsetse fly-human contacts. The factor *c*_2_ represents the effort of treatment to control the disease, and the factor *c*_3_ also represents the effort of using insecticides to ensure that the breeding sites of the tsetse fly are minimized. Hence, taking into account the assumptions and extensions made, we try to find the most effective strategy that reduces the HAT infection in the population at a very minimum cost. With the use of bounded Lebesgue measurable control, we define the objective function to be minimized as

(40) $$\begin{array}{c} J\left(c_{1},c_{2},c_{3}\right)=\int_{0}^{t_{F}}\left(M_{1}e_{\text{h}}+M_{2}i_{\text{h}}+M_{3}e_{\text{v}}+M_{4}i_{\text{v}}+\frac{1}{2}k_{1}c_{1}^{2}+\frac{1}{2}k_{2}c_{2}^{2}+\frac{1}{2}k_{3}c_{3}^{2}\right)\;dt. \end{array}$$

Thus, the dynamics of the controls that minimizes the objective function is given by

(41) $$\begin{array}{c} \left\{\begin{array}{c} \frac{ds_{\text{h}}}{dt}=\pi_{\text{h}}+\rho r_{\text{h}}-\left(1-c_{1}\right)ap_{\text{h}}i_{\text{v}}s_{\text{h}}-\mu_{\text{h}}s_{\text{h}},\\ \frac{de_{\text{h}}}{dt}=\left(1-c_{1}\right)ap_{\text{h}}i_{\text{v}}s_{\text{h}}-\varepsilon e_{\text{h}}-\mu_{\text{h}}e_{\text{h}},\\ \frac{di_{\text{h}}}{dt}=\varepsilon e_{\text{h}}-\mu_{\text{h}}i_{\text{h}}-\omega i_{\text{h}}-c_{2}\tau i_{\text{h}},\\ \frac{dr_{\text{h}}}{dt}=c_{2}\tau i_{\text{h}}-\rho r_{\text{h}}-\mu_{\text{h}}r_{\text{h}},\\ \frac{ds_{\text{v}}}{dt}=\pi_{\text{v}}-c_{3}\mu_{\text{v}}s_{\text{v}}-\left(1-c_{1}\right)ap_{\text{v}}i_{\text{h}}s_{\text{v}},\\ \frac{de_{\text{v}}}{dt}=\left(1-c_{1}\right)ap_{\text{v}}i_{\text{h}}s_{\text{v}}-c_{3}\mu_{\text{v}}e_{\text{v}}-\sigma e_{\text{v}},\\ \frac{di_{\text{v}}}{dt}=\sigma e_{\text{v}}-c_{3}\mu_{\text{v}}i_{\text{v}}, \end{array}\right. \end{array}$$

subject to the initial conditions *s*_h_ ≥ 0, *e*_h_ ≥ 0, *i*_h_ ≥ 0, *r*_h_ ≥ 0, *s*_v_ ≥ 0, *e*_v_ ≥ 0, and  *i*_v_ ≥ 0. The associated effective reproduction number for (41) denoted by *R*_E_ is obtained as

(42) $$\begin{array}{c} R_{\text{E}}=\sqrt{\frac{a^{2}\varepsilon {\left(1-c_{1}\right)}^{2}p_{\text{h}}\pi_{\text{h}}\pi_{\text{v}}\sigma }{c_{3}\mu_{\text{h}}\left(\varepsilon +\mu_{\text{h}}\right)\mu_{\text{v}}^{2}\left(\sigma +c_{3}\mu_{\text{v}}\right)\left(\mu_{\text{h}}+c_{2}\tau +\omega \right)}}=\sqrt{R_{c}}. \end{array}$$

The goal is to minimize the exposed and infectious human populations (*e*_h_, *i*_h_), the exposed and infectious vector populations (*e*_v_, *i*_v_), and the cost of implementing the control by the use of possible *c*_*i*_,  *i*=1, 2, 3. The functional objective includes the social cost which relates to the resources that are needed for educating people on personal protection (1/2)*k*_1_ *c*_1_^2^, the application of treatment (1/2)*k*_2_ *c*_2_^2^, and spraying of tsetse fly operations (1/2)*k*_3_ *c*_3_^2^. The quantities *M*_1_ and *M*_2_, respectively, represent the associated cost with minimizing the exposed and infected human population, while *M*_3_ and *M*_4_ also represent the cost associated with minimizing the exposed and infected vectors, respectively. The quantity *t*_F_ is the time period of intervention. As explained in [15], the costs corresponding to *M*_1_ *e*_h_, *M*_2_ *i*_h_, *M*_3_ *e*_v_, and *M*_4_ *i*_v_ are linear, while the cost control functions (1/2)*k*_1_ *c*_1_^2^, (1/2)*k*_2_ *c*_2_^2^, and (1/2)*k*_3_ *c*_3_^2^ should be nonlinear and take a quadratic form. Therefore, we seek to minimize the objective function over the given time interval [0, *t*_F_]. Pontryagin's maximum principle is used to solve this optimal control problem and the derivation of the necessary conditions. The Lagrangian of the optimal control problem is given by

(43) $$\begin{array}{c} L=\left(M_{1}e_{\text{h}}+M_{2}i_{\text{h}}+M_{3}e_{\text{v}}+M_{4}i_{\text{v}}+\frac{1}{2}k_{1}c_{1}^{2}+\frac{1}{2}k_{2}c_{2}^{2}+\frac{1}{2}k_{3}c_{3}^{2}\right). \end{array}$$

To determine the Lagrangian minimum value, we define the Hamiltonian, *H*, for the control problem as

(44) $$\begin{array}{c} H=M_{1}e_{\text{h}}+M_{2}i_{\text{h}}+M_{3}e_{\text{v}}+M_{4}i_{\text{v}}+\frac{1}{2}k_{1}c_{1}^{2}+\frac{1}{2}k_{2}c_{2}^{2}+\frac{1}{2}k_{3}c_{3}^{2}+\lambda_{s_{\text{h}}}\frac{ds_{\text{h}}}{dt}+\lambda_{e_{\text{h}}}\frac{de_{\text{h}}}{dt}+\lambda_{i_{\text{h}}}\frac{di_{\text{h}}}{dt}+\lambda_{r_{\text{h}}}\frac{dr_{\text{h}}}{dt}+\lambda_{s_{\text{v}}}\frac{ds_{\text{v}}}{dt}+\lambda_{e_{\text{v}}}\frac{de_{\text{v}}}{dt}+\lambda_{i_{\text{v}}}\frac{di_{\text{v}}}{dt}, \end{array}$$

where *λ*_*s*_*h*__, *λ*_*e*_*h*__, *λ*_*i*_*h*__, *λ*_*r*_*h*__, *λ*_*s*_*v*__, *λ*_*e*_*v*__, and  *λ*_*i*_*v*__ are adjoint variables or costate variables. The differential equations of adjoint variables are obtained by taking the partial derivatives of the Hamiltonian equation with respect to the state variables, which gives

(45) $$\begin{array}{c} \left\{\begin{array}{c} \frac{d\lambda_{s_{\text{h}}}}{dt}=\lambda_{s_{\text{h}}}\mu_{h}+\left(\lambda_{s_{\text{h}}}-\lambda_{e_{\text{h}}}\right)\left(1-c_{1}\right)ap_{\text{h}}i_{\text{v}},\\ \frac{d\lambda_{e_{\text{h}}}}{dt}=-M_{1}+\left(\lambda_{e_{\text{h}}}-\lambda_{i_{\text{h}}}\right)\varepsilon +\lambda_{e_{\text{h}}}\mu_{\text{h}},\\ \frac{d\lambda_{i_{\text{h}}}}{dt}=-M_{2}+\left(\lambda_{i_{\text{h}}}-\lambda_{r_{\text{h}}}\right)c_{2}\tau +\lambda_{i_{\text{h}}}\left(\omega +\mu_{\text{h}}\right)+\left(\lambda_{s_{\text{v}}}-\lambda_{e_{\text{v}}}\right)\left(1-c_{1}\right)ap_{\text{v}}s_{\text{v}},\\ \frac{d\lambda_{r_{\text{h}}}}{dt}=\left(\lambda_{r_{\text{h}}}-\lambda_{s_{\text{h}}}\right)\rho +\lambda_{r_{\text{h}}}\mu_{\text{h}},\\ \frac{d\lambda_{s_{\text{v}}}}{dt}=\left(\lambda_{s_{\text{v}}}-\lambda_{e_{\text{v}}}\right)\left(1-c_{1}\right)ap_{\text{v}}i_{\text{h}}+\lambda_{s_{\text{v}}}\mu_{\text{v}}c_{3},\\ \frac{d\lambda_{e_{\text{v}}}}{dt}=-M_{3}+\left(\lambda_{e_{\text{v}}}-\lambda_{i_{\text{v}}}\right)\sigma +\lambda_{e_{\text{v}}}\mu_{\text{v}}c_{3},\\ \frac{d\lambda_{i_{\text{v}}}}{dt}=-M_{4}+\left(\lambda_{s_{\text{h}}}-\lambda_{e_{\text{h}}}\right)\left(1-c_{1}\right)ap_{\text{h}}s_{\text{h}}+\lambda_{i_{\text{v}}}\mu_{\text{v}}c_{3}. \end{array}\right. \end{array}$$

**Theorem 6 .**

Given the optimal controls *c*_1_^*∗*^, *c*_2_^*∗*^, *c*_3_^*∗*^ and the solutions *s*_h_, *e*_h_, *i*_h_, *r*_h_, *s*_v_, *e*_v_, *i*_v_ of the corresponding state equations (41) and (40) which minimize *J*(*c*_1_, *c*_2_, *c*_3_) over the region Ω, then there exist adjoint variables *λ*_*s*_h__, *λ*_*e*_h__, *λ*_*i*_h__, *λ*_*r*_h__, *λ*_*s*_v__, *λ*_*e*_v__, *λ*_*i*_v__ satisfying

(46) $$\begin{array}{c} -\frac{d\lambda_{i}}{dt}=\frac{\partial H}{\partial i},\;i\in \left\{s_{\text{h}},e_{\text{h}},i_{\text{h}},r_{\text{h}},s_{\text{v}},e_{\text{v}},i_{\text{v}}\right\}, \end{array}$$

and the optimal solution *c*_1_^*∗*^, *c*_2_^*∗*^, *c*_3_^*∗*^ is given by

(47) $$\begin{array}{c} c_{1}^{*}=\min\left\{1,\max\left(0,\hat{c_{1}}\right)\right\},\\ c_{2}^{*}=\min\left\{1,\max\left(0,\hat{c_{2}}\right)\right\},\\ c_{3}^{*}=\min\left\{1,\max\left(0,\hat{c_{3}}\right)\right\}. \end{array}$$

*Proof*. The Pontryagin's maximum principle described in [16, 17] is applied. Corollary 4.1 in [17] shows the existence of an optimal control due to the convexity of the integrand *J* with respect to *c*_1_, *c*_2_, *c*_3_, and Lipschitz property of the state system with respect to the state variables. By using the optimal conditions

(48) $$\begin{array}{c} \frac{\partial H}{\partial c_{1}}=0,\\ \frac{\partial H}{\partial c_{2}}=0,\\ \frac{\partial H}{\partial c_{3}}=0, \end{array}$$

we obtain,

(49) $$\begin{array}{c} \left\{\begin{array}{c} \frac{\partial H}{\partial c_{1}}=k_{1}c_{1}+\left(\lambda_{s_{\text{h}}}-\lambda_{e_{\text{h}}}\right)ap_{\text{h}}i_{\text{v}}s_{\text{h}}+\left(\lambda_{s_{\text{v}}}-\lambda_{e_{\text{v}}}\right)ap_{\text{v}}i_{\text{h}}s_{\text{v}}=0,\\ \frac{\partial H}{\partial c_{2}}=k_{2}c_{2}+\left(\lambda_{r_{\text{h}}}-\lambda_{i_{\text{h}}}\right)\tau i_{\text{h}}=0,\\ \frac{\partial H}{\partial c_{3}}=k_{3}c_{3}-\left(\lambda_{s_{\text{v}}}\mu_{\text{v}}s_{\text{v}}+\lambda_{e_{\text{v}}}\mu_{\text{v}}e_{\text{v}}+\lambda_{i_{\text{v}}}\mu_{\text{v}}i_{\text{v}}\right)=0. \end{array}\right. \end{array}$$

Solving (49), we have

(50) $$\begin{array}{c} \hat{c_{1}}=\frac{\left(\lambda_{e_{\text{h}}}-\lambda_{s_{\text{h}}}\right)ap_{\text{h}}i_{\text{v}}s_{\text{h}}+\left(\lambda_{e_{\text{v}}}-\lambda_{s_{\text{v}}}\right)ap_{\text{v}}i_{\text{h}}s_{\text{v}}}{k_{1}},\\ \hat{c_{2}}=\frac{\left(\lambda_{i_{\text{h}}}-\lambda_{r_{\text{h}}}\right)\tau i_{\text{h}}}{k_{2}},\\ \hat{c_{3}}=\frac{\lambda_{s_{v}}\mu_{\text{v}}s_{\text{v}}+\lambda_{e_{\text{v}}}\mu_{\text{v}}e_{\text{v}}+\lambda_{i_{\text{v}}}\mu_{\text{v}}i_{v}}{k_{3}}. \end{array}$$

As stated earlier, the lower and upper boundaries for the control parameters are 0 and 1, respectively. If $\hat{c_{1}},\hat{c_{2}},\hat{c_{3}}<1$, then *c*_1_=*c*_2_=*c*_3_=0 and if $\hat{c_{1}},\hat{c_{2}},\hat{c_{3}}>1$, then *c*_1_=*c*_2_=*c*_3_=1, otherwise $c_{1}=\hat{c_{1}},c_{2}=\hat{c_{2}},c_{3}=\hat{c_{3}}$. Therefore, for the control parameters *c*_1_^*∗*^, *c*_2_^*∗*^, *c*_3_^*∗*^, we obtain the optimum value of the function *J*(*c*_1_, *c*_2_, *c*_3_).

### 4.1. Optimal Control Simulations

The Octave programming language is used to simulate the optimal control model using the set of parameters obtained from previously reported studies and datasets, which have been cited. Some of these parameters are assumed for the sake of illustrations.  Table 2 represents the values of the model parameters used for simulations. The following initial conditions were considered:

(51) $$\begin{array}{c} s_{\text{h}}\left(0\right)=30,\\ e_{\text{h}}\left(0\right)=7,\\ i_{\text{h}}\left(0\right)=2,\\ r_{\text{h}}\left(0\right)=0,\\ s_{\text{v}}\left(0\right)=40,\\ e_{\text{v}}\left(0\right)=10,\\ i_{\text{v}}\left(0\right)=3, \end{array}$$

and the weight constants were assumed to be

(52) $$\begin{array}{c} M_{1}=1,\\ M_{2}=2,\\ M_{3}=2,\\ M_{4}=2,\\ k_{1}=2,\\ k_{2}=10,\\ k_{3}=5. \end{array}$$

**Figure 2:**
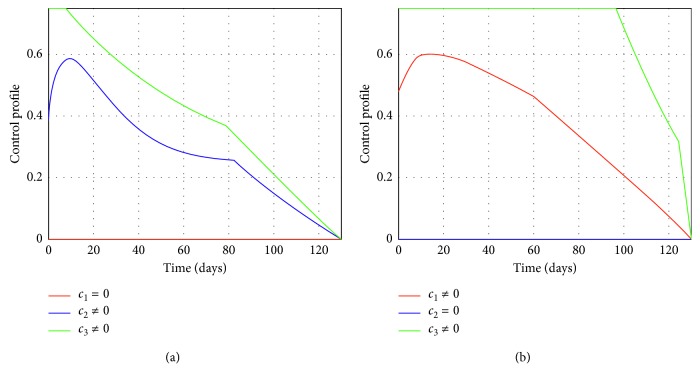
(a) Control profile when *c*_1_=0, *c*_2_ ≠ 0, and *c*_3_ ≠ 0 at *R*_*c*_ = 0.0075. (b) Control profile when *c*_1_ ≠ 0, *c*_2_=0, and *c*_3_ ≠ 0 at *R*_*c*_ = 0.00013.

**Figure 3 fig3:**
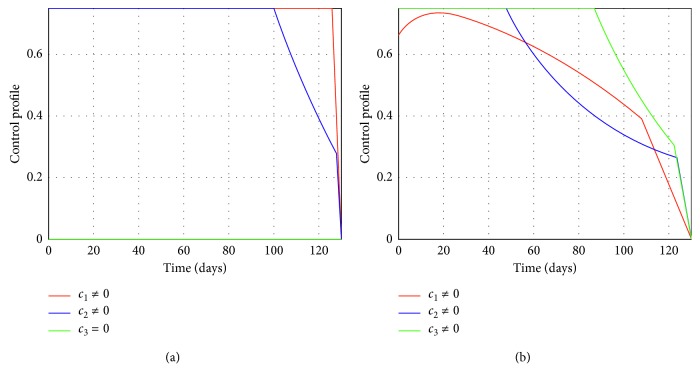
(a) Control profile when *c*_1_ ≠ 0, *c*_2_ ≠ 0, and *c*_3_=0 at *R*_*c*_ = 0.044. (b) Control profile when *c*_1_ ≠ 0, *c*_2_ ≠ 0, and *c*_3_ ≠ 0 at *R*_*c*_ = 0.000075.

**Figure 4 fig4:**
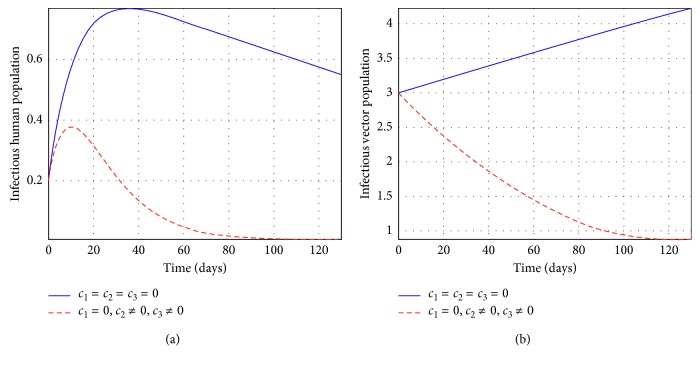
Simulations of the model showing the efforts of treatment and insecticides only on infectious individuals at *R*_*c*_ = 0.0075.

**Figure 5 fig5:**
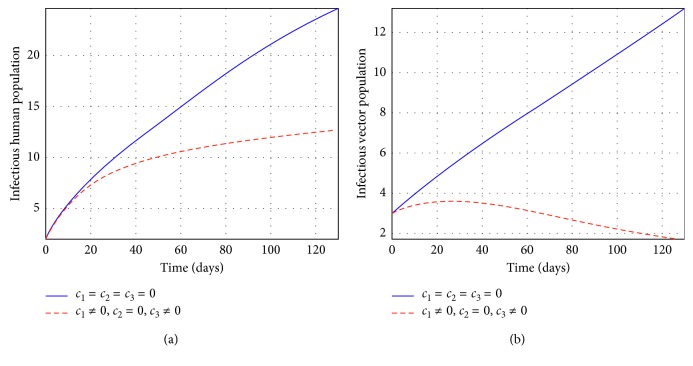
Simulations of the model showing the efforts of education and insecticides only on infectious individuals at *R*_*c*_ = 0.00013.

**Figure 6 fig6:**
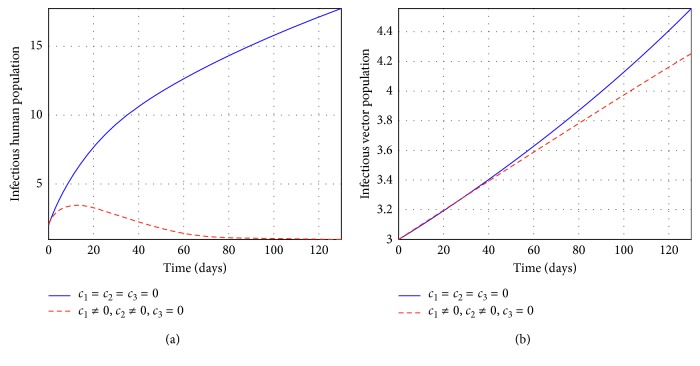
Simulations of the model showing the efforts of education and treatment only on infectious individuals at *R*_*c*_ = 0.044.

**Figure 7:**
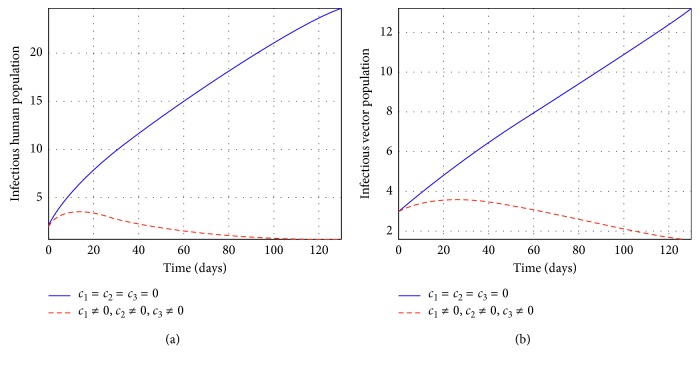
Simulations of the model showing the efforts of education, treatment, and insecticides on infectious individuals at *R*_*c*_ = 0.000075.

Figures 2 and 3 represent the control profiles at different values of *c*_1_, *c*_2_, and *c*_3_, while the rest of the plots are the graphs of infectious human and vector population plotted against time in days and they represent the effect of optimal controls *c*_1_, *c*_2_, and *c*_3_ in reducing the number of individuals infected. From Figure 4, we observe that the use of treatment and insecticides only has a significant impact in reducing the number of infectious individuals and they show that this strategy is effective to control tsetse flies and infected human populations. In Figure 5, we observe that the use of education and insecticides reduce the number of infectious individuals but the results depicted in Figure 5(a) shows that this strategy is not effective and efficient to control the infectious human population. From Figure 6, we observe that the use of education and treatment reduces the number of infectious individuals but the results from Figure 6(b) shows that this strategy is not effective and efficient to control the population of infectious tsetse flies. Lastly, the results depicted from Figure 7 shows that the strategy of using education, treatment, and insecticides is very efficient and effective to reduce the number of infected individuals. Therefore, the use of education, treatment, and insecticides simultaneously is very efficient and effective to eliminate HAT in Africa.

In epidemiology, a reproduction number less than unity implies that the disease can be eradicated in the long run. Hence, choosing suitable parameters for the controls *c*_1_, *c*_2_, and *c*_3_, it was observed that the effective reproduction numbers obtained for Figures 2 [Fig fig3] [Fig fig4] [Fig fig5] [Fig fig6]–7 were 0.0075, 0.00013, 0.044, 0.000075, 0.0075, 0.00013, 0.044, and 0.000075, respectively. This shows that incorporating all the control measures, that is, educating individuals, giving treatment, and applying insecticides, is an effective method to help reduce the number secondary infections in the population which corresponds with eradicating the disease in the long run.

## 5. Conclusion

In this paper, we studied and analyzed the model for transmission of HAT, and determined the basic reproduction number. The local and global stabilities of disease-free equilibrium and endemic equilibrium were also proved. For the optimal control model, education, treatment, and insecticides as control measures were used to optimize the objective function defined by Equation (40). The numerical simulations of the optimal control model show that the best strategy to reduce the number of infected individuals is through the use of education, treatment, and insecticides. This is the effective and efficient method to eliminate the disease. Furthermore, the national authorities, nongovernmental organizations (NGOs), and stakeholders must not lose their interest in controlling the disease because neglecting this disease may cause rapid reoccurrence and much effect to the people who are at risk.

## Figures and Tables

**Table 1:** The description of model variables and parameters.

Variable	Description
*s*_h_	Susceptible human population
*s*_v_	Susceptible tsetse fly population
*e*_h_ and *e*_v_	Exposed human and tsetse fly population, respectively
*i*_h_ and *i*_v_	Infectious human and tsetse population, respectively
*r*_h_	Recovered human population

Parameter	Description

*π*_h_	Recruitment rate for human population
*π*_v_	Recruitment rate for tsetse fly population
*p*_h_	Proportion of bites by the infectious vector on susceptible human population
*p*_v_	Proportion of bites by susceptible vector on an infectious human population
*a*	The biting rate of the tsetse flies
*σ*	Per capita rate of a vector becoming infectious
*ε*	Per capita rate of human becoming infectious
*ω*	Disease induced death rate
*ρ*	The rate at which the recovered human can become susceptible again
*τ*	Recovery rate
*μ*	Natural death rate
*ξ*_h_	Force of infection for human population
*ξ*_v_	Force of infection for tsetse flies

**Table 2:** Parameters values used for simulations.

Parameter	Value	Reference
*π*_h_	0.000215/day	[14]
*π*_v_	0.07/day	[14]
*p*_h_	0.62	[6]
*p*_v_	0.065	[6, 15]
*a*	Varying	Assumed
*σ*	0.001	Assumed
*ε*	0.083	[18]
*ω*	0.004	[3]
*ρ*	0.02	[6]
*τ*	0.125	[3]
*μ*_h_	0.00044	Assumed
*μ*_v_	0.034	[15]

## Data Availability

The secondary data supporting this research are from previously reported studies and datasets, which have been cited. The processed data are available in cited references.
